# Nonspecific Signs and/or Symptoms of Cancer: A Retrospective, Observational Analysis from a Secondary Care, US Community Oncology Dataset

**DOI:** 10.3390/curroncol31070268

**Published:** 2024-06-25

**Authors:** Christopher B. Benton, Ding He, Karen Todoroff, Marie V. Coignet, Ying Luan, Jason C. Wong, Kathryn N. Kurtzman, Ira Zackon

**Affiliations:** 1 Rocky Mountain Cancer Centers, Englewood, CO 80113, USA; 2 Ontada, Boston, MA 02109, USA; 3 GRAIL, LLC, Menlo Park, CA 94025, USA; mcoignet@grailbio.com (M.V.C.);

**Keywords:** nonspecific signs and/or symptoms, cancer diagnosis, early detection

## Abstract

To help determine the unmet need for improved diagnostic tools to evaluate patients with nonspecific signs and/or symptoms (NSSS) and suspicion of cancer, we examined patient characteristics, diagnostic journey, and cancer incidence of patients with NSSS within The US Oncology Network (The Network), a secondary care community oncology setting. This retrospective, observational cohort study included patients aged ≥40 years with ≥1 NSSS in their problem list at their first visit within The Network (the index date) between 1 January 2016 and 31 December 2020. Patients were followed longitudinally with electronic health record data for initial cancer diagnosis, new noncancer diagnosis, death, end of study observation period, or 12 months, whichever occurred first. Of 103,984 patients eligible for inclusion, 96,722 presented with only 1 NSSS at index date; 6537/103,984 (6.3%) were diagnosed with 1 primary cancer within 12 months after the index date; 3825/6537 (58.5%) with hematologic malignancy, and 2712/6537 (41.5%) with solid tumor. Among patients diagnosed with cancer (n = 6774), the median time to cancer diagnosis after their first visit within The Network was 5.13 weeks. This study provides a real-world perspective on cancer incidence in patients with NSSS referred to a secondary care setting and highlights the unmet need for improved diagnostic tools to improve cancer outcomes.

## 1. Introduction

Approximately 1.8 million people are diagnosed with cancer and 600,000 people die from cancer in the United States (US) each year [1]. Earlier diagnosis has been linked with reduced mortality, reduced healthcare costs, and improved outcomes for multiple cancer types by providing more effective, less expensive treatments and earlier care [2,3,4]. More than 60% of cancers in the US are diagnosed following symptomatic presentation [5]. While some symptoms are specific for certain cancers (e.g., breast lump for breast cancer and rectal bleeding for colorectal cancer) and suggest a clear diagnostic evaluation pathway, nonspecific signs and/or symptoms such as weight loss, anemia, fatigue, and pain could be associated with a range of cancer and noncancer diagnoses and therefore have no clear diagnostic evaluation pathway [6]. This may cause patients to undergo undirected or misdirected diagnostic evaluation while the possibility of cancer and search for its origin are explored. Those with cancer may have a delayed diagnosis, delayed treatment, and poorer outcomes [2,7,8], while those without cancer are often subjected to various unnecessary procedures due to initial cancer suspicion. Additionally, patients with cancer who present with nonspecific signs and/or symptoms are more likely to be diagnosed through emergency presentation and have a prolonged time to diagnosis than those who present with site-specific signs and/or symptoms [9,10].

In Europe, there are various national programs set up to expedite diagnostic evaluation for nonspecific signs and/or symptoms. Denmark has established the Non-specific Symptoms and Signs of Cancer Patient Pathway (NSSC-CPP), a pathway for patients with serious nonspecific signs and/or symptoms of cancer (e.g., weight loss, fatigue, pain, and nausea), which consists of two steps directed by primary care physicians (PCPs) [11,12]. Step 1 includes blood and urine tests, and if there is continued suspicion of cancer, Step 2 includes either X-ray and ultrasound of abdomen or computed tomography scan of the thorax, abdomen, and pelvis. In England, based on the Danish system, rapid diagnostic clinics are being expanded nationally by the National Health Service [13]. These rapid diagnostic clinics are for the triage of patients without site-specific cancer symptoms. In contrast, a national diagnostic pathway for suspected cancer does not exist in the US. Although more than 50% of oncology practices in the US report use of clinical pathways to streamline treatment [14], standardized diagnostic pathways are not common. PCPs typically own referral and investigative decisions and could benefit from convenient, noninvasive tools that can direct diagnostic evaluation of nonspecific signs and/or symptoms and help achieve an earlier diagnosis. In fact, such tools could also improve existing cancer diagnostic pathways and clinics in Europe.

The US Oncology Network (The Network) is a national network of over 500 community-based oncology practices. Patients are typically referred to a physician in The Network by their PCP. In this retrospective, observational cohort study, we used data from The Network to examine characteristics, diagnostic journey, and cancer incidence of patients with nonspecific signs and/or symptoms in secondary care with the objective of determining the unmet need for improved diagnostic processes and tools for cancer in patients with nonspecific signs and/or symptoms. 

## 2. Materials and Methods

### 2.1. Data Source

Structured data among patients within The Network were extracted from the iKnowMed (iKM; McKesson Corporation, Irving, TX, USA) electronic health records (EHR; the iKM EHR captures outpatient practice encounter histories for patients under community-based care within The Network) and the Financial Database Warehouse (FDW; repository of healthcare claims submitted by practices within The Network). The Network provides care for over 1.2 million patients annually across 500 cancer treatment center locations across the US. 

The study was approved by the McKesson Compliance/Privacy and Institutional Review Board (protocol 22-005E). The study was performed in accordance with the Declaration of Helsinki. Because this study involves analysis of secondary data and all data are deidentified, exemption status under 45 CFR 46.104(d)(4)(iii) and a waiver of informed consent under 45 CFR 164.512(i)(2)(ii) were granted by the Institutional Review Board.

### 2.2. Study Cohort

This retrospective, observational cohort study included patients aged ≥40 years, with ≥1 nonspecific signs and/or symptoms in their EHR problem list at their first visit within The Network after referral (the index date), and with ≥1 additional visit within 12 months after the index date. The identification period was from 1 January 2016, to 31 December 2020. Nonspecific signs and/or symptoms were defined as anemia, venous thromboembolism, general malaise, weight loss, nonspecific abdominal symptoms, new and unexplained breathlessness, unexplained worsening pain, abnormal lab test results, abnormal liver function test, abnormal coagulation profile, abnormal iron, and abnormal platelet or white blood cell (WBC) count [13]. Patients were excluded if diagnosed with any cancer (except nonmetastatic basal cell and squamous cell carcinoma skin cancer) within 3 years on or prior to the index date. 

### 2.3. Outcomes and Statistical Analysis

Patient demographics, clinical characteristics, clinical outcomes (time to cancer and noncancer diagnosis), and healthcare resource utilization (HCRU) were recorded. Patients were followed longitudinally with data from EHRs for initial cancer diagnosis, new noncancer diagnosis, and death until the end of study observation period (31 December 2021) or end of diagnostic evaluation period (at 12 months), whichever occurred first. Diagnoses were collected up to 12 months after the index date. 

Patients could have multiple cancer and noncancer conditions diagnosed at one or more visits. Patients diagnosed with cancer could have also been diagnosed with noncancer conditions. Among patients not diagnosed with cancer, the first noncancer diagnosis (ascertained using International Classification of Diseases [ICD]-10 code categories) that occurred during the follow-up period was captured; these patients were defined as patients diagnosed with noncancer. Cancer diagnoses were ascertained through a review of iKM’s discrete diagnosis and histology fields, which were populated during the routine course of care (ICD codes were not used). Cancer diagnoses were categorized as the following: hematologic (lymphoid leukemia, lymphoma, myeloid neoplasm, plasma cell neoplasm); upper gastrointestinal (liver/bile duct, esophageal, gallbladder, gastrointestinal stromal tumors, pancreatic, small intestine, stomach [gastric]); lower gastrointestinal (anal, colon, rectal, appendix); breast; genitourinary (bladder, kidney [renal cell], penile, prostate, testicular, ureteral); gynecologic (cervical, endometrial, fallopian tube, ovarian, primary peritoneal, uterine, vaginal, vulvar); head and neck (hypopharyngeal, laryngeal, lip and oral cavity, metastatic squamous neck cancer with occult primary, nasopharyngeal, oropharyngeal, paranasal sinus and nasal cavity, pharyngeal, salivary gland); respiratory (non-small-cell lung cancer, small-cell lung cancer, malignant mesothelioma); melanoma; and other solid tumors. Time to cancer and noncancer diagnosis were calculated from the index date until date of diagnosis. HCRU was captured using Current Procedural Terminology (CPT) codes. 

Outcomes were assessed descriptively. Categorical variables were reported as counts (percentage), and continuous variables were reported as mean ± standard deviation (SD).

## 3. Results

### 3.1. Patient Disposition

Among the 232,559 patients with ≥1 nonspecific signs and/or symptoms in their problem list at their first visit within The Network during the identification period, 170,874 were aged ≥40 years at index date and had ≥1 additional visit within 12 months after the index date (Table 1). Of these, 66,872 were excluded for having a documented diagnosis of any cancer (except for basal cell carcinoma and squamous cell carcinoma skin cancer) in the EHR at index date or within the previous 3 years. An additional 18 patients were excluded for having inconsistent data. The final cohort consisted of 103,984 patients.

### 3.2. Patient Demographics

The mean (SD) age of patients was 64.9 (13.4) years, and 53,875 (51.8%) patients were aged ≥ 65 years. A total of 67,004 (64.4%) patients were female; 67,154 (64.6%) were White; 48,321 (46.5%) were never smokers; 42,099 (40.5%) were obese (body mass index ≥ 30); and 50,115 (48.2%) were from a southern practice region in the US. The mean (SD) follow-up time from the index date until date of death, last visit, or end of study period, whichever occurred first, was 20.0 (17.7) months. A total of 76,202 (73.3%) patients were seen by a hematologist/medical oncologist during the study observation period. Detailed patient demographics overall and by nonspecific signs and/or symptoms subgroups are shown in Table 2.

### 3.3. Clinical Characteristics

**Figure 1:**
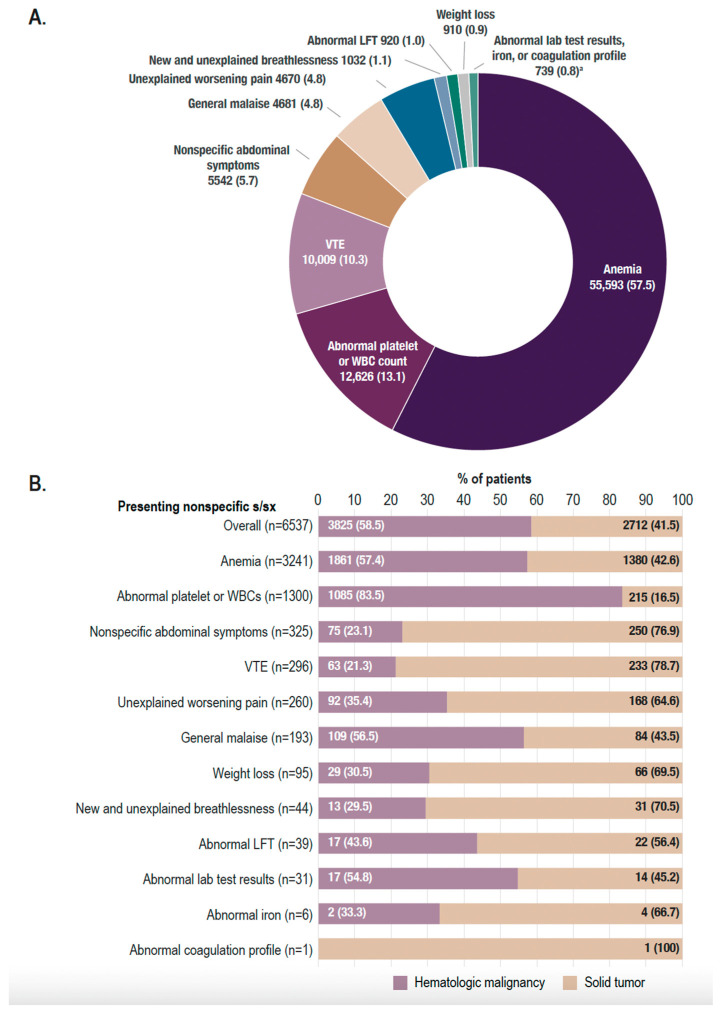
Presenting nonspecific signs and/or symptoms and primary cancer type distribution in patients with only 1 presenting nonspecific sign and/or symptom. (**A**) Number (%) of patients with each prespecified nonspecific sign and/or symptom. (**B**) Among patients diagnosed with 1 primary cancer (n = 6537), number (%) of patients diagnosed with a primary hematologic malignancy or primary solid tumor, stratified by the presenting nonspecific signs and/or symptoms. See Table S1 for a breakdown of the diagnosed primary cancer types stratified by presenting nonspecific signs and/or symptoms. ^a^ Abnormal lab test results, 537 (0.6%) patients; abnormal iron, 149 (0.2%) patients; abnormal coagulation profile, 53 (0.1%) patients. LFT, liver function test; s/sx, signs and/or symptoms; VTE, venous thromboembolism; WBC, white blood cell.

Among patients with only 1 presenting nonspecific sign and/or symptom at index date (n = 96,722), the most common (in ≥10% of patients) presenting nonspecific signs and/or symptoms were anemia (55,593 [57.5%] patients), abnormal platelet or WBC count (12,626 [13.1%]), and venous thromboembolism (10,009 [10.3%]) (Figure 1A).

### 3.4. Clinical Outcomes

A total of 6774/103,984 (6.5%) patients were diagnosed with cancer. When stratified by the presenting nonspecific signs and/or symptoms, the proportion of patients diagnosed with cancer was highest in patients presenting with only weight loss (11.2%; Table 3). A total of 6537/6774 (96.5%) patients were diagnosed with 1 primary cancer. Among patients diagnosed with 1 primary cancer, 3825 (58.5%) were diagnosed with a primary hematologic malignancy and 2712 (41.5%) with a solid tumor (Table 4); the most common (in ≥5% of patients) primary solid tumor diagnoses were lower gastrointestinal (470 [7.2%] patients), genitourinary (412 [6.3%]), respiratory (395 [6.0%]), upper gastrointestinal (373 [5.7%]), and breast (350 [5.4%]) cancer. Primary cancer diagnoses based on presenting nonspecific signs and/or symptoms are shown in Figure 1B. Notably, a number of nonspecific signs and/or symptoms (nonspecific abdominal symptoms, venous thromboembolism, unexplained worsening pain, weight loss, new and unexplained breathlessness, abnormal liver function tests, abnormal iron, and abnormal coagulation profile) were primarily associated with solid tumors.

A total of 38,718/103,984 (37.2%) patients were diagnosed with noncancer conditions. Of these, 28,955 (74.8%) patients had only 1 noncancer diagnosis date. The most common (in ≥10% of patients) noncancer conditions in patients with only 1 noncancer diagnosis date were blood and blood-forming organ diseases and certain disorders involving immune mechanism (18,134 [62.6%] patients); circulatory system diseases (6413 [22.1%]); endocrine, nutritional and metabolic diseases (5326 [18.4%]); and musculoskeletal system and connective tissue diseases (2971 [10.3%]). Patients could have more than one noncancer diagnosis on the same date.

### 3.5. Time to Diagnosis in Secondary Care

**Figure 2:**
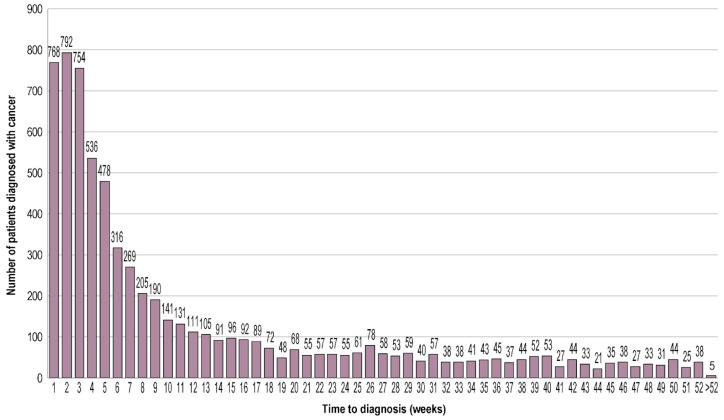
Time from the index date to new cancer diagnosis. Time to diagnosis (weeks) was calculated from the index date (date patient presents with a nonspecific sign and/or symptom in their problem list at their first visit within The US Oncology Network after referral from a primary care physician) to new cancer diagnosis. Patients with a time to diagnosis >52 weeks had a cancer diagnosis on day 365 after the index date, which is 1 day greater than 52 weeks.

Among patients diagnosed with cancer (n = 6774), the median time from first visit within The Network with nonspecific signs and/or symptoms to new cancer diagnosis was 5.13 weeks (solid: 7.12 weeks; hematologic: 4.41 weeks); by 17 and 34 weeks, 5164 (76.2%) and 6099 (90.0%) patients with cancer received a diagnosis, respectively (Figure 2).

Among patients diagnosed with only 1 noncancer diagnosis date (n = 28,955), by 1 week, 23,509 (81.2%) patients received a noncancer diagnosis. A similar result was observed among patients with >1 noncancer diagnosis date (n = 9763); 8253 (84.5%) patients received their first noncancer diagnosis in 1 week.

### 3.6. Healthcare Resource Utilization

HCRU in patients diagnosed with cancer and with noncancer are summarized in Table S2. Among patients with HCRU records, patients diagnosed with cancer (n = 6732) compared with patients diagnosed with noncancer (n = 38,524) had higher use of lab tests (5814 [86.4%] vs. 24,636 [63.9%]), outpatient services (5477 [81.4%] vs. 24,497 [63.6%]), biopsies (1487 [22.1%] vs. 496 [1.3%]), and imaging (545 [8.1%] vs. 2453 [6.4%]).

## 4. Discussion

We examined the patient characteristics, diagnostic journey, and cancer incidence of patients with nonspecific signs and/or symptoms within The Network, a secondary care community oncology setting across the US. We structured our nonspecific signs and/or symptoms according to those of the English rapid diagnostic clinics [13], which encompass signs and/or symptoms of potential concern for cancer. The patient population was approximately half <65 years of age, majority female, and a minority was non-White, obese, and never smokers. Our study provides a valuable descriptor of patients with nonspecific signs and/or symptoms and suspicion of cancer in the US.

Nonspecific signs and/or symptoms present a complex challenge in timely and cost-effective cancer diagnoses. An important key observation in this study was that the incidence of cancer was higher in this study population with nonspecific signs and/or symptoms (6.5%) compared to the general US population aged ≥40 years (1.0%) [15]. Among the nonspecific signs and/or symptoms studied, weight loss was associated with the highest proportion of cancer diagnoses (11.2%). This proportion is slightly higher than that previously reported in a retrospective cohort analysis of primary care EHR data from the US (7.8%) [16]; the difference could be attributed to different clinical settings—secondary care in the current study versus primary care in the previous study. While there may be some selection bias toward cancer diagnoses in this study—given that patients were referred to a secondary care community oncology setting—there remains concern of potential delayed cancer diagnosis in the general population due to the ambiguity of nonspecific signs and/or symptoms. Secondly, it was notable that while the majority of cancers diagnosed were primary hematologic malignancies (reflecting referral patterns to a hematologist/medical oncologist), a substantial proportion of patients within this population with a cancer diagnosis were ultimately diagnosed with primary solid tumors within 1 year (~40%). This trend may be attributed to the fact that 42.6% of patients diagnosed with cancer who presented with only anemia (the most common presenting signs and/or symptoms in this study) were diagnosed with a primary solid tumor. At the same time, only 6.0% of patients presenting with only anemia were diagnosed with cancer. Moreover, only 3.1% of patients presenting with only venous thromboembolism were diagnosed with cancer, giving some real-world perspective on the association of malignancy and hypercoagulable state. Most solid tumors present with a broad range of signs and/or symptoms, including ‘alarm’ symptoms (features in a patient’s presentation that help predict malignant disease) [17] as well as nonspecific signs and/or symptoms [6]. For example, rectal bleeding, an alarm symptom, and anemia and abdominal pain, nonspecific signs and/or symptoms, are all common presenting symptoms for colorectal cancer [6,18,19]. 

The median time to cancer diagnosis in this study was 5.13 weeks, and it took 17 weeks and 34 weeks for 75% and 90% of patients with cancer to reach a diagnosis, respectively. The fact that time to diagnosis for solid tumors (7.12 weeks) was longer than that for hematologic cancers (4.41 weeks) could potentially indicate that the association of solid tumors with nonspecific signs and/or symptoms presents more challenging diagnostic evaluations, with an incongruous symptom–diagnosis axis, leading to longer time to diagnosis. It is important to note that the median time to diagnosis in this study was measured from the start of secondary care instead of the first PCP visit to final diagnosis. The median time to cancer diagnosis could be considerably longer when starting from the first PCP visit. For example, in a study of 153 patients diagnosed with primary lung cancer in England using primary care and hospital records from 2010 to 2012, the median time from first signs and/or symptoms (including alarm symptoms) to diagnosis was 13 weeks [20]. In another study of 3329 patients diagnosed with hematologic malignancy in the United Kingdom using patient surveys from 2004 to 2011, the median time from help-seeking due to symptom (including alarm symptoms) to diagnosis ranged from 1.3 to 26.4 weeks depending on the cancer type [21]. As such, although the time to cancer diagnosis was on the lower end in our study given the secondary care setting, ~25% and ~10% of patients still required >17 and >34 weeks to reach diagnosis, respectively.

Patients diagnosed with cancer had a numerically higher HCRU than patients diagnosed with noncancer, consistent with reasonable expectations. However, a limitation of this study is that it only captures HCRU within The Network. HCRU associated with physician consultations or diagnostic tests performed outside The Network are not captured; therefore, the reported HCRU does not reflect the complete HCRU for patients with nonspecific signs and/or symptoms. As a result, similar to time to diagnosis, HCRU is an underestimation in this study; this impacts our understanding of the costs of diagnostics in the majority of patients presenting with nonspecific signs and/or symptoms who do not have cancer. 

Given the lack of standardization and thus limitations of existing cancer diagnostic pathways, effective tools capable of aiding the diagnostic evaluation of nonspecific signs and/or symptoms by stratifying risk for cancer across multiple cancer types may be valuable to achieve timely and accurate cancer or noncancer diagnosis and reduce the risk of an extended diagnostic evaluation and potential harm from undirected or misdirected diagnostic procedures. In the US, where PCPs are generally the first to see patients with nonspecific signs and/or symptoms and manage subsequent diagnostic evaluation, such tools could have value in directing diagnostic evaluation procedures to achieve timely diagnosis and treatment to optimize outcomes. Potential tools include using primary care blood tests (albumin, alkaline phosphatase, C-reactive protein, hemoglobin, liver enzymes, platelets, and total white cell count) in patients with nonspecific signs and/or symptoms [22] or using prediction algorithms to identify which patients may require further investigation for cancer [23,24]. Additionally, multi-cancer detection tests, which analyze tumor-derived analytes that differentiate cancer from noncancer, are one potential advancement to provide decision support and cancer risk stratification in symptomatic patients to reduce diagnostic delays [25,26]. For example, the SYMPLIFY study (ISRCTN10226380) clinically validated the performance of a methylation-based multi-cancer detection test (initially designed and validated as a screening test) [27] in symptomatic patients referred from primary care for urgent cancer investigation in the UK [28]. 

This study has a number of limitations. First, there may be additional nonspecific signs and/or symptoms among patients within The Network that may not have explicitly been captured in The Network iKM EHR. Second, the study is unable to make correlations of cancer diagnosis with cancer stage, as stage of cancer diagnoses was not captured in the structured data. Third, this study defined the end of the diagnostic evaluation period as 12 months in order to associate nonspecific signs and/or symptoms with cancer diagnoses; thus, any diagnoses at or beyond 12 months from the index date were excluded from our analysis. Fourth, we have limited capacity to differentiate between new cancer diagnoses and recurrences. However, the exclusion of patients with existing invasive cancer diagnoses within 3 years prior should minimize the likelihood of capturing cancer recurrences. Finally, the retrospective and observational nature of the study design precludes making robust correlations between nonspecific signs and/or symptoms and diagnoses (cancer and noncancer).

## 5. Conclusions

In our study of patients with nonspecific signs and/or symptoms, referred to a secondary care community oncology setting, we observed a relatively high incidence of cancer, including a range of hematologic malignancies and solid tumors. The time to cancer diagnosis was longer than optimal and is likely underestimated from the duration of symptoms preceding referral. Along with the lack of cancer diagnostic pathways, our study provides a real-world perspective on the incidence of cancer diagnoses in patients with nonspecific signs and/or symptoms referred to a secondary care community oncology setting and highlights the unmet need for improved diagnostic tools that may stratify risk for cancer and assist in achieving more timely and cost-effective management of these patients to improve cancer outcomes while avoiding costly undirected or misdirected testing in the majority with benign conditions.

## Figures and Tables

**Table 1:** Patient attrition.

Inclusion/Exclusion Criteria	Patients Remaining
Patients with ≥1 nonspecific signs and/or symptoms from their iKM EHR problem list within The US Oncology Network between 1 January 2016, and 31 December 2020	251,704
Above visit is their first visit for nonspecific signs and/or symptoms between 1 January 2016, and 31 December 2020	232,559
Patients ≥40 years of age at visit with nonspecific signs and/or symptoms (index date)	201,163
Patients ≥1 additional vital visit within 12 months after the index visit	170,874
Exclude patients with a documented diagnosis of any cancer (excluding basal cell carcinoma and squamous cell carcinoma skin cancer) in iKM EHR within 3 years on or prior to the index date	104,002
Exclude patients with erroneous data	103,984

iKM, iKnowMed; EHR, electronic health records; US, United States.

**Table 2:** Patient demographics.

	Overall (N = 103,984)	Patients with Only 1 Presenting Nonspecific Sign and/or Symptom (n = 96,722)											
		Anemia (n = 55,593)	Abnormal Platelet or WBC Counts (n = 12,626)	VTE (n = 10,009)	Nonspecific Abdominal Symptoms (n = 5542)	General Malaise (n = 4681)	Unexplained Worsening Pain (n = 4670)	New/Unexplained Breathlessness (n = 1032)	Abnormal Liver Function Test (n = 920)	Weight Loss (n = 910)	Abnormal Lab Test Results (n = 537)	Abnormal Iron (n = 149)	Abnormal Coagulation Profile (n = 53)
Age													
Mean (SD)	64.9 (13.4)	65.5 (14.0)	64.7 (12.8)	62.6 (12.2)	64.2 (12.0)	64.9 (12.9)	64.6 (11.9)	67.1 (11.8)	61.8 (11.3)	69.4 (12.1)	63.0 (12.0)	62.5 (12.0)	61.3 (12.5)
≥65 years, n (%)	53,875 (51.8)	30,273 (54.5)	6364 (50.4)	4329 (43.3)	2694 (48.6)	2339 (50.0)	2309 (49.4)	597 (57.8)	369 (40.1)	597 (65.6)	239 (44.5)	63 (42.3)	25 (47.2)
Sex, n (%)													
Female	67,004 (64.4)	38,143 (68.6)	6912 (54.7)	5176 (51.7)	3897 (70.3)	3069 (65.6)	3051 (65.3)	652 (63.2)	522 (56.7)	495 (54.4)	334 (62.2)	99 (66.4)	31 (58.5)
Race, n (%)													
White	67,154 (64.6)	34,054 (61.3)	8811 (69.8)	6419 (64.1)	3981 (71.8)	3450 (73.7)	3281 (70.3)	769 (74.5)	654 (71.1)	595 (65.4)	363 (67.6)	91 (61.1)	36 (67.9)
Black	14,788 (14.2)	9516 (17.1)	1016 (8.0)	1391 (13.9)	453 (8.2)	466 (10.0)	472 (10.1)	78 (7.6)	47 (5.1)	149 (16.4)	79 (14.7)	13 (8.7)	3 (5.7)
Asian	2305 (2.2)	1238 (2.2)	344 (2.7)	77 (0.8)	143 (2.6)	63 (1.3)	123 (2.6)	37 (3.6)	47 (5.1)	14 (1.5)	17 (3.2)	11 (7.4)	2 (3.8)
Native American	297 (0.3)	146 (0.3)	39 (0.3)	11 (0.1)	33 (0.6)	18 (0.4)	18 (0.4)	3 (0.3)	2 (0.2)	0 (0.00)	1 (0.2)	0 (0.00)	0 (0.00)
Other	2350 (2.3)	1346 (2.4)	265 (2.1)	186 (1.9)	104 (1.9)	74 (1.6)	112 (2.4)	24 (2.3)	23 (2.5)	20 (2.2)	9 (1.7)	14 (9.4)	2 (3.8)
Not documented	17,090 (16.4)	9293 (16.7)	2151 (17.0)	1925 (19.2)	828 (14.9)	610 (13.0)	664 (14.2)	121 (11.7)	147 (16.0)	132 (14.5)	68 (12.7)	20 (13.4)	10 (18.9)
Practice region, n (%) ^a^													
South	50,115 (48.2)	29,674 (53.4)	4801 (38.0)	4276 (42.7)	2035 (36.7)	2828 (60.4)	1862 (39.9)	367 (35.6)	220 (23.9)	355 (39.0)	348 (64.8)	11 (7.4)	9 (17.0)
West	30,749 (29.6)	13,368 (24.0)	4974 (39.4)	3172 (31.7)	2311 (41.7)	988 (21.1)	2066 (44.2)	419 (40.6)	567 (61.6)	246 (27.0)	127 (23.7)	131 (87.9)	40 (75.5)
Midwest	17,212 (16.6)	9434 (17.0)	2005 (15.9)	1795 (17.9)	964 (17.4)	645 (13.8)	579 (12.4)	195 (18.9)	94 (10.2)	233 (25.6)	37 (6.9)	3 (2.0)	1 (1.9)
Northeast	5908 (5.7)	3117 (5.6)	846 (6.7)	766 (7.7)	232 (4.2)	220 (4.7)	163 (3.5)	51 (4.9)	39 (4.2)	76 (8.4)	25 (4.7)	4 (2.7)	3 (5.7)
Smoking status, n (%)													
Never	48,321 (46.5)	26,725 (48.1)	5082 (40.3)	5042 (50.4)	2590 (46.7)	2083 (44.5)	1939 (41.5)	444 (43.0)	455 (49.5)	357 (39.2)	187 (34.8)	76 (51.0)	24 (45.3)
Former	30,323 (29.2)	16,481 (29.6)	3567 (28.3)	2875 (28.7)	1648 (29.7)	1226 (26.2)	1238 (26.5)	364 (35.3)	306 (33.3)	300 (33.0)	101 (18.8)	48 (32.2)	16 (30.2)
Current	8637 (8.3)	3797 (6.8)	1754 (13.9)	699 (7.0)	471 (8.5)	365 (7.8)	467 (10.0)	74 (7.2)	71 (7.7)	165 (18.1)	45 (8.4)	17 (11.4)	6 (11.3)
Not documented	16,703 (16.1)	8590 (15.5)	2223 (17.6)	1393 (13.9)	833 (15.0)	1007 (21.5)	1026 (22.0)	150 (14.5)	88 (9.6)	88 (9.7)	204 (38.0)	8 (5.4)	7 (13.2)
BMI (kg/m^2^) group, n (%)													
Obese (BMI ≥ 30)	42,099 (40.5)	22,334 (40.2)	5238 (41.5)	4988 (49.8)	1850 (33.4)	1770 (37.8)	1838 (39.4)	384 (37.2)	341 (37.1)	113 (12.4)	230 (42.8)	47 (31.5)	29 (54.7)
Overweight (BMI = 25–29.9)	29,549 (28.4)	15,564 (28.0)	3690 (29.2)	2712 (27.1)	1707 (30.8)	1449 (31.0)	1415 (30.3)	297 (28.8)	297 (32.3)	215 (23.6)	165 (30.7)	42 (28.2)	11 (20.8)
Normal (BMI = 18.5–24.9)	23,766 (22.9)	13,081 (23.5)	2761 (21.9)	1293 (12.9)	1590 (28.7)	1140 (24.4)	1096 (23.5)	262 (25.4)	220 (23.9)	442 (48.6)	107 (19.9)	45 (30.2)	10 (18.9)
Underweight (BMI < 18.5)	1469 (1.4)	777 (1.4)	149 (1.2)	50 (0.5)	124 (2.2)	70 (1.5)	63 (1.3)	16 (1.6)	12 (1.3)	70 (7.7)	5 (0.9)	3 (2.0)	0 (0.00)
Not Documented	7101 (6.8)	3837 (6.9)	788 (6.2)	966 (9.7)	271 (4.9)	252 (5.4)	258 (5.5)	73 (7.1)	50 (5.4)	70 (7.7)	30 (5.6)	12 (8.1)	3 (5.7)
Months of follow-up													
Mean (SD)	20.0 (17.7)	19.9 (17.6)	18.7 (19.0)	19.8 (17.8)	22.0 (18.0)	21.6 (15.6)	23.2 (17.0)	22.5 (17.9)	23.4 (19.8)	17.2 (16.0)	19.0 (16.8)	20.3 (19.0)	16.6 (20.5)

^a^ The US census region of the clinic where the patient received care at the index visit. South was defined as Delaware, Florida, Georgia, Maryland, North Carolina, South Carolina, Virginia, Washington D.C., West Virginia, Alabama, Kentucky, Mississippi, Tennessee, Arkansas, Louisiana, Oklahoma, and Texas. West was defined as Arizona, Colorado, Idaho, Montana, Nevada, New Mexico, Utah, Wyoming, California, Oregon, and Washington State. Midwest was defined as Illinois, Indiana, Michigan, Ohio, Wisconsin, Iowa, Kansas, Minnesota, Missouri, Nebraska, North Dakota, and South Dakota. Northeast was defined as Connecticut, Maine, Massachusetts, New Hampshire, Rhode Island, Vermont, Pennsylvania, New Jersey, and New York. BMI, body mass index; SD, standard deviation; VTE, venous thromboembolism; WBC, white blood cell.

**Table 3:** Proportion of patients with only 1 presenting nonspecific sign and/or symptom diagnosed with cancer.

	Number of Patients Presenting with Only 1 Nonspecific Sign and/or Symptom	Number of Patients Diagnosed with Cancer	Proportion of Patients Diagnosed with Cancer
Anemia	55,593	3363	6.0%
Abnormal platelet or WBC counts	12,626	1329	10.5%
VTE	10,009	309	3.1%
Nonspecific abdominal symptoms	5542	337	6.1%
General malaise	4681	203	4.3%
Unexplained worsening pain	4670	275	5.9%
New/unexplained breathlessness	1032	45	4.4%
Abnormal liver function test	920	40	4.3%
Weight loss	910	102	11.2%
Abnormal lab test results	537	33	6.1%
Abnormal iron	149	6	4.0%
Abnormal coagulation profile	53	1	1.9%

**Table 4:** Distribution of primary cancer diagnoses.

	Patients Diagnosed with 1 Primary Cancer (n = 6537)
Primary hematologic malignancy, n (%)	3825 (58.5)
Myeloid neoplasm ^a^	1911 (28.2)
Lymphoma	1147 (16.9)
Plasma cell neoplasm	678 (10)
Lymphoid leukemia	89 (1.3)
Primary solid tumor, n (%)	2712 (41.5)
Lower gastrointestinal	470 (7.2)
Genitourinary	412 (6.3)
Respiratory	395 (6.0)
Upper gastrointestinal	373 (5.7)
Breast	350 (5.4)
Other ^b^	301 (4.6)
Gynecologic	296 (4.5)
Melanoma	66 (1.0)
Head and neck	49 (0.7)

^a^ Acute myeloid leukemia or chronic myeloid leukemia. ^b^ The Other category includes endocrine, neuroendocrine, neurologic, sarcoma, thyroid, nonmelanoma skin cancer, other (unclassified), and unknown primary.

## Data Availability

The datasets used and/or analyzed during the current study may be shared by the corresponding author (Ira Zackon, ira.zackon@usoncology.com) upon request.

## Supplementary Materials

The following supporting information can be downloaded at: https://www.mdpi.com/article/10.3390/curroncol31070268/s1, Table S1: Primary cancer type distribution; Table S2: Healthcare research utilization.
