# Supplementary material for: Nonspecific Signs and/or Symptoms of Cancer: A Retrospective, Observational Analysis from a Secondary Care, US Community Oncology Dataset
Source: Curr Oncol. 2024 Jun 25;31(7):3643–56. doi: 10.3390/curroncol31070268 (PMC11276305; doi:10.3390/curroncol31070268)
Supplement: Supplementary file 1 [file curroncol-31-00268-s001.zip › curroncol-3027876-supplementary.pdf]

Supplemental Results

Table S1. Primary cancer type distribution.

Primary cancer diagnosis	Overall (n = 6537)	Patients with Only 1 Presenting Nonspecific Sign and/or Symptom											
		Anemia (n = 3241)	Abnormal Platelet or WBCs (n = 1300)	Nonspecific Abdominal Symptoms (n = 325)	VTE (n = 296)	Unexplained Worsening Pain (n = 260)	General Malaise (n = 193)	Weight Loss (n = 95)	New and Unexplained Breathlessness (n = 44)	Abnormal LFT (n = 39)	Abnormal Lab Test Results (n = 31)	Abnormal Iron (n = 6)	Abnormal Coagulation Profile (n = 1)
Hematologic malignancy, n (%)	3825 (58.5)	1861 (57.4)	1085 (83.5)	75 (23.1)	63 (21.3)	92 (35.4)	109 (56.5)	29 (30.5)	13 (29.5)	17 (43.6)	17 (54.8)	2 (33.3)	0
Myeloid neoplasm [a]	1911 (29.2)	949 (29.3)	604 (46.5)	17 (5.2)	20 (6.8)	15 (5.8)	38 (19.7)	9 (9.5)	7 (15.9)	9 (23.1)	3 (9.7)	1 (16.7)	0
Lymphoma	1147 (17.5)	420 (13.0)	417 (32.1)	40 (12.3)	32 (10.8)	34 (13.1)	42 (21.8)	14 (14.7)	1 (2.3)	5 (12.8)	2 (6.5)	1 (16.7)	0
Plasma cell neoplasm	678 (10.4)	464 (14.3)	30 (2.3)	16 (4.9)	9 (3.0)	42 (16.2)	26 (13.5)	5 (5.3)	5 (11.4)	3 (7.7)	12 (38.7)	0	0
Lymphoid leukemia	89 (1.4)	28 (0.9)	34 (2.6)	2 (0.6)	2 (0.7)	1 (0.4%)	3 (1.6)	1 (1.1)	0	0	0	0	0
Solid tumor, n (%)	2712 (41.5)	1380 (42.6)	215 (16.5)	250 (76.9)	233 (78.7)	168 (64.6)	84 (43.5)	66 (69.5)	31 (70.5)	22 (56.4)	14 (45.2)	4 (66.7)	1 (100)
Lower gastrointestinal	470 (7.2)	332 (10.2)	24 (1.8)	26 (8.0)	26 (8.8)	8 (3.1)	9 (4.7)	4 (4.2)	1 (2.3)	1 (2.6)	0	1 (16.7)	0
Genitourinary	412 (6.3)	224 (6.9)	33 (2.5)	20 (6.2)	36 (12.2)	32 (12.3)	10 (5.2)	6 (6.3)	3 (6.8)	4 (10.3)	3 (9.7)	0	0
Respiratory	395 (6.0)	180 (5.6)	32 (2.5)	21 (6.5)	31 (10.5)	40 (15.4)	16 (8.3)	22 (23.2)	14 (31.8)	0	3 (9.7)	1 (16.7)	1 (100)
Upper gastrointestinal	373 (5.7)	153 (4.7)	30 (2.3)	50 (15.4)	29 (9.8)	18 (6.9)	15 (7.8)	17 (17.9)	0	12 (30.8)	2 (6.5)	1 (16.7)	0
Breast	350 (5.4)	199 (6.1)	38 (2.9)	5 (1.5)	41 (13.9)	22 (8.5)	11 (5.7)	3 (3.2)	2 (4.5)	3 (7.7)	3 (9.7)	1 (16.7)	0
Other [b]	301 (4.6)	119 (3.7)	35 (2.7)	29 (8.9)	26 (8.8)	22 (8.5)	15 (7.8)	10 (10.5)	8 (18.2)	2 (5.1)	1 (3.2)	0	0
Gynecologic	296 (4.5)	114 (3.5)	13 (1.0)	93 (28.6)	29 (9.8)	20 (7.7)	3 (1.6)	3 (3.2)	2 (4.5)	0	1 (3.2)	0	0
Melanoma	66 (1.0)	36 (1.1)	4 (0.3)	2 (0.6)	9 (3.0)	4 (1.5)	3 (1.6)	1 (1.1)	0	0	1 (3.2)	0	0
Head and neck	49 (0.7)	23 (0.7)	6 (0.5)	4 (1.2)	6 (2.0)	2 (0.8)	2 (1.0)	0	1 (2.3)	0	0	0	0

[a] Acute myeloid leukemia or chronic myeloid leukemia. [b] Other category includes endocrine, neuroendocrine, neurologic, sarcoma, thyroid, non-melanoma skin cancer, other (unclassified), and unknown primary. s/sx, sign and/or symptom; VTE, venous thromboembolism; WBC, white blood cell.

Table S2. Healthcare research utilization.

	Patients Diagnosed with Cancer with HCRU Records [a] (n = 6732)	Patients Diagnosed with Noncancer with HCRU Records [b] (n = 38,524)
Lab tests		
Patients with ≥1, n (%)	5814 (86.4)	24,636 (63.9)
Mean number per patient (SD)	15.5 (18.4)	10.7 (24.4)
Outpatient services		
Patients with ≥1, n (%)	5477 (81.4)	24,497 (63.6)
Mean number per patient (SD)	2.0 (2.2)	1.6 (2.8)
Biopsies		
Patients with ≥1, n (%)	1487 (22.1)	496 (1.3)
Mean number per patient (SD)	0.2 (0.5)	0 (0.1)
Imaging		
Patients with ≥1, n (%)	545 (8.1)	2453 (6.4)
Mean number per patient (SD)	0.2 (1.9)	0.2 (1.5)

[a] During the follow-up period from index date until initial cancer diagnosis (up to 12 months). [b] During the fixed 12-month follow-up period from index date. HCRU, health care resource utilization; SD, standard deviation.
